# The Role of Omega-3 Fatty Acid Supplementation in Slowing Cognitive Decline Among Elderly Patients With Alzheimer's Disease: A Systematic Review of Randomized Controlled Trials

**DOI:** 10.7759/cureus.73390

**Published:** 2024-11-10

**Authors:** Gaurav Vijayrao Deshmukh, Humaira Niaz, Riya Bai, Dong Hwi Kim, Ji Woo Kim, Jawaria Asghar, Taha Ramzan, Muhammad Maqbool, Nada B Abushalha, Sidra Arif, Safdar Khan

**Affiliations:** 1 Special Newborn Care Unit, District Hospital, Ahmednagar, IND; 2 Internal Medicine, Peshawar Medical College, Peshawar, PAK; 3 Internal Medicine, Chandka Medical College, Larkana, PAK; 4 Internal Medicine, Pusan National University, Yangsan, KOR; 5 Faculty of Medicine Health and Human Sciences, Macquarie University, Sydney, AUS; 6 Internal Medicine, Basic Health Unit Jamal Pur, Gujrat, PAK; 7 Medical Education, Services Institute of Medical Sciences, Lahore, PAK; 8 Internal Medicine, Shaheed Mohtarma Benazir Bhutto Medical College Lyari, Karachi, PAK; 9 Gynecology, The University of Jordan, Amman, JOR; 10 Urology, Jinnah Postgraduate Medical Centre, Karachi, PAK; 11 Surgery, Services Hospital Lahore, Lahore, PAK

**Keywords:** alzheimer's disease, cognitive decline, docosahexaenoic acid, mild cognitive impairment, omega-3 fatty acids

## Abstract

This systematic review explores the impact of omega-3 fatty acid supplementation, particularly docosahexaenoic acid (DHA), on cognitive decline in individuals with mild cognitive impairment (MCI) and Alzheimer’s disease (AD). Omega-3 fatty acids are widely recognized for their neuroprotective properties, but the evidence regarding their efficacy in mitigating cognitive decline remains mixed. Through a comprehensive analysis of eleven randomized controlled trials, we aimed to assess the role of DHA in improving cognitive functions and slowing brain atrophy. The findings revealed that DHA supplementation demonstrated cognitive benefits, particularly in memory and hippocampal volume preservation, in some studies involving early-stage cognitive decline, while others reported negligible effects, particularly in more advanced Alzheimer’s disease. The review identified variations in study design, dosage, intervention duration, and population characteristics as potential factors contributing to the inconsistencies observed across trials. Despite these mixed outcomes, DHA’s safety profile and potential for early intervention in at-risk populations offer promise for its use in clinical practice. This review underscores the need for further longitudinal, large-scale studies to refine DHA dosage recommendations, optimize intervention timing, and explore personalized approaches based on genetic factors. The insights gained from this review contribute to a growing understanding of the role omega-3 fatty acids could play in managing cognitive decline and highlight future directions for research.

## Introduction and background

Omega-3 fatty acids, particularly docosahexaenoic acid (DHA), have garnered considerable attention in neurodegenerative disease research due to their purported neuroprotective effects [1,2]. DHA is the most abundant omega-3 fatty acid in the brain, where it plays a critical role in maintaining neuronal structure and function. Its neuroprotective properties are thought to stem from several mechanisms, including the regulation of synaptic plasticity, the reduction of neuroinflammation, and the enhancement of neuronal membrane fluidity [3]. These effects may contribute to improved signal transmission and neurogenesis, as well as the inhibition of apoptosis, which could collectively slow the progression of cognitive decline in conditions such as Alzheimer’s disease (AD) and mild cognitive impairment (MCI) [4,5]. Emerging evidence from epidemiological and animal studies supports DHA’s involvement in mitigating cognitive decline, further justifying its exploration in clinical trials.

Despite the promising role of DHA, variability among studies examining its cognitive benefits in AD and MCI populations remains significant. Differences in study design, dosage, intervention duration, and participant characteristics have led to inconsistent findings regarding the efficacy of DHA supplementation in improving cognitive function and reducing brain atrophy. This systematic review critically examines randomized controlled trials (RCTs) to address these discrepancies, aiming to provide a clearer understanding of DHA’s potential role in managing cognitive decline in elderly populations diagnosed with AD or MCI.

The primary objective of this systematic review was to synthesize and evaluate the evidence from randomized controlled trials assessing the impact of omega-3 fatty acid supplementation, specifically DHA, on cognitive functions and hippocampal volume in older adults with MCI and AD. Given the variability in outcomes and methodologies across different studies, this review aims to provide a clear, comprehensive understanding of the extent to which omega-3 fatty acids can benefit individuals suffering from these cognitive disorders. The guiding research question for this review is: "Does supplementation with omega-3 fatty acids, particularly DHA, improve cognitive function and reduce hippocampal atrophy in elderly patients with mild cognitive impairment or Alzheimer’s disease?" This question seeks to clarify the therapeutic potential and dosage efficacy of omega-3 fatty acids in clinical settings, contributing to the broader discourse on non-pharmacological interventions in the management of cognitive decline.

## Review

Materials and methods

Search Strategy

Our search methodology was rigorously crafted in compliance with the preferred reporting items for systematic reviews and meta-analyses (PRISMA) guidelines [6] to assess the efficacy of omega-3 fatty acid supplementation, particularly docosahexaenoic acid (DHA), in slowing cognitive decline in older adults with mild cognitive impairment (MCI) and Alzheimer’s disease (AD). To encompass a comprehensive spectrum of pertinent literature, we performed exhaustive searches across multiple prominent electronic databases, including PubMed, Medline, Embase, the Cochrane Library, and Scopus. The search timeframe spanned from the inception of each database up to September 2024, ensuring the inclusion of both historical and recent developments in the field.

Keywords and medical subject headings (MeSH) were meticulously selected to refine and optimize the search, focusing on terms directly relevant to our research question. These terms included "omega-3 fatty acids," "docosahexaenoic acid," "cognitive decline," "mild cognitive impairment," "Alzheimer’s disease," and "randomized controlled trials." Boolean operators ('AND', 'OR') were utilized to structure and expand the search strategy effectively. Example search strings employed were "omega-3 fatty acids AND cognitive decline AND randomized controlled trials," "DHA supplementation AND Mild Cognitive Impairment," and "Alzheimer’s disease AND omega-3 fatty acids OR DHA AND clinical outcomes." To extend the reach of our search and capture additional relevant studies, we reviewed the reference lists of all included articles and searched clinical trial registries as well as pertinent conference proceedings for unpublished or ongoing studies. This comprehensive approach, reviewed by a medical information specialist with expertise in neurodegenerative diseases, aimed to ensure the thoroughness and precision of our search strategy, capturing a wide array of data to answer our defined research question effectively.

Eligibility Criteria

The eligibility criteria for this systematic review have been meticulously delineated to ensure the inclusion of high-quality, relevant studies that investigate the efficacy of omega-3 fatty acids, particularly docosahexaenoic acid (DHA), in enhancing cognitive function and decelerating hippocampal atrophy in elderly individuals with mild cognitive impairment (MCI) and Alzheimer’s disease (AD). Our review focuses on peer-reviewed research articles, including randomized controlled trials (RCTs), clinical trials, and meta-analyses that address the cognitive outcomes associated with omega-3 or DHA supplementation in specified populations. We include studies that are specifically designed to evaluate cognitive function and brain structural changes, employing well-defined and validated measurement tools and methods. The inclusion criteria stipulate that eligible studies must be published in English in peer-reviewed journals, from the inception of the respective databases until September 2024, to integrate both seminal and contemporary research findings.

Exclusion criteria were established to refine the scope of our review and exclude studies that did not meet the specific objectives and methodological standards required for a rigorous analysis. Studies that did not focus on the administration of omega-3 fatty acids or DHA or did not target elderly populations diagnosed with MCI or AD were excluded to maintain a clear focus on the intended subject population and intervention. Additionally, studies lacking randomized controlled or clinical trial designs were omitted to ensure the inclusion of evidence with the highest possible methodological rigor. We also excluded non-English studies to avoid the complexities and potential inaccuracies associated with translation. Lastly, studies that did not provide sufficient detail on the intervention protocols, dosages, duration, and specific cognitive or volumetric outcomes were excluded to preserve the scientific integrity and applicability of our systematic review findings.

Data Extraction

Our data extraction protocol was rigorously structured to capture and validate crucial information from selected studies on the impact of omega-3 fatty acid supplementation on cognitive decline in elderly patients with MCI or AD. Following the initial article screening based on titles and abstracts, two independent reviewers assessed the relevance of each article, categorizing them into "relevant," "not relevant," or "potentially relevant" categories. Articles advancing to full-text review were scrutinized using a predefined, standardized data extraction sheet in Microsoft Excel to ensure uniformity and accuracy across evaluations. Any discrepancies between reviewers were resolved through consultation with a third, senior reviewer, ensuring a consistent and objective approach to data inclusion and analysis.

Data Analysis and Synthesis

Our data analysis and synthesis approach was carefully constructed to ensure a robust assessment of the effects of omega-3 fatty acid supplementation on cognitive decline in patients with MCI or AD. Using a structured, systematic review methodology, we extracted data from each study into a predefined template in Microsoft Excel (Redmond, USA) to facilitate a comprehensive comparative analysis. This template included variables such as study author, publication year, study design, sample size, intervention details (type and dosage of omega-3 fatty acids), cognitive outcomes, and any noted limitations or biases. Quantitative data were analyzed using meta-analytical techniques, where appropriate, to compute effect sizes, with heterogeneity assessed through I^2 statistics and publication bias examined via funnel plots. Qualitative synthesis was performed when the meta-analysis was not feasible and summarized findings narratively to delineate the impact of omega-3 fatty acid supplementation on cognitive metrics across diverse study populations and settings.

Results

Study Selection Process

The study selection process for this systematic review followed a structured approach to ensure the inclusion of relevant, high-quality studies, as defined by methodological rigor and relevance to the research question. Initially, 149 records were identified from multiple databases (PubMed, Medline, Embase, the Cochrane Library, and Scopus), with 18 duplicate records removed before screening. The remaining 131 records were screened based on their titles and abstracts, during which 37 were excluded for not meeting the inclusion criteria. Reasons for exclusion at this stage primarily included irrelevance to omega-3 fatty acid supplementation, non-randomized study designs, and studies not involving populations diagnosed with MCI or AD.

Out of 94 reports sought for full-text retrieval, 30 were not available due to restricted access or incomplete information, leaving 64 reports for eligibility assessment. Following a comprehensive full-text review, 54 reports were excluded for several reasons, including inadequate sample size (e.g., fewer than 50 participants), study design not meeting the inclusion criteria (e.g., non-randomized or observational studies), inconsistent or poorly defined outcome measures (e.g., using non-standardized cognitive assessments), insufficient detail on intervention protocols, dosages, or duration of DHA supplementation, and lack of relevant cognitive outcomes, such as memory function or brain atrophy. Ultimately, 10 studies were included in the final review, providing a robust basis for analyzing the effects of omega-3 fatty acids on cognitive decline.

**Figure 1:**
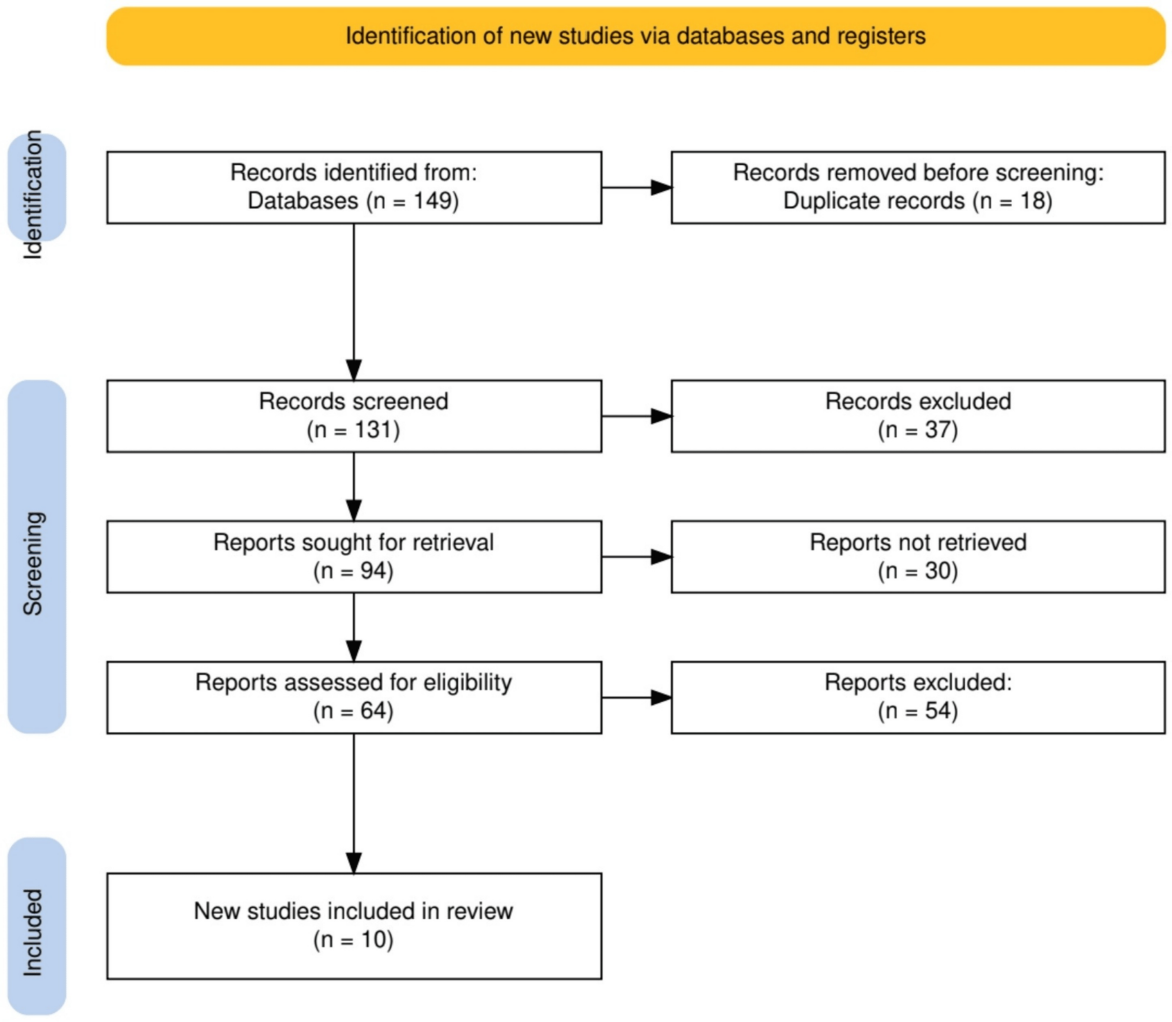
The PRISMA flow diagram illustrates the study selection process. PRISMA: Preferred reporting items for systematic reviews and meta-analyses

To assess the quality of the included studies, we employed the Cochrane Risk of Bias Tool. This tool evaluates key domains such as randomization methods, allocation concealment, blinding of participants and personnel, completeness of outcome data, and selective reporting. Studies were categorized as low, moderate, or high risk of bias, and only those classified as low or moderate risk were included in the final analysis to ensure methodological rigor. This rigorous process ensured that only high-quality studies, characterized by sound design and reliable outcome measures, contributed to the evidence base for this systematic review (Figure 1).

Characteristics of the Selected Studies

The selected studies for this systematic review include a range of randomized, double-blind, placebo-controlled trials that evaluate the effects of omega-3 fatty acids, particularly DHA, on cognitive function in populations with MCI and AD. These studies span durations from four months to three years and involve diverse participant populations, including healthy elderly individuals, those with MCI, and individuals with mild to moderate cognitive impairment. The interventions primarily involved DHA supplementation at varying dosages, with outcome measures focusing on cognitive function (such as ADAS-cog, MMSE), brain atrophy, and biochemical markers like Aβ levels and cerebrospinal fluid (CSF) DHA concentrations. While some studies reported significant improvements in memory and cognitive function, especially in early-stage cognitive decline, others found no significant benefit in more advanced Alzheimer’s disease.

In addition to summarizing the key aspects of the selected studies, we have also incorporated a detailed risk of bias assessment using the Cochrane Risk of Bias Tool. This evaluation considered factors such as randomization methods, allocation concealment, blinding of participants and personnel, incomplete outcome data, and selective reporting. These assessments provide a critical layer of analysis, ensuring that the findings from the included studies are interpreted with a clear understanding of potential biases. The combination of study characteristics, outcomes, and the risk of bias evaluation offers a comprehensive overview of the mixed results observed in this research field. Below is a detailed table summarizing the key aspects and risk of bias assessments of the selected studies (Table 1).

**Table 1:** Summary of key characteristics and findings of randomized controlled trials discussed in the article. DHA: Docosahexaenoic acid, g/d: grams per day, ADAS-cog: Alzheimer's disease assessment scale – cognitive subscale, CDR: Clinical dementia rating, ARCD: Age-related cognitive decline, CANTAB: Cambridge neuropsychological test automated battery, PAL: Paired associate learning, CIND: Cognitive impairment no dementia, PUFA: Polyunsaturated fatty acids, EPA: Eicosapentaenoic acid, CAIDE: Cardiovascular risk factors, aging, and dementia, FCSRT: Free and cued selective reminding test, MMSE: Mini-mental state examination, CSF: Cerebrospinal fluid, MRI: Magnetic resonance imaging, APOE4: Apolipoprotein E ε4 allele, MCI: Mild cognitive impairment, Aβ: Amyloid-beta, FA: Fatty acids, CIBIC-plus: Clinician’s interview-based impression of change plus caregiver input

Study Reference	Study Type	Duration	Participants	Intervention	Outcome Measures	Results	Conclusion	Risk of Bias Assessment
Quinn JF et al., 2010 [7]	Randomized, double-blind, placebo-controlled trial	18 months	Mild to moderate Alzheimer disease	Algal DHA 2 g/d or placebo	ADAS-cog, CDR, brain atrophy	No significant effect of DHA on cognitive or functional decline, or brain atrophy.	DHA does not slow cognitive or functional decline in Alzheimer disease.	Low risk: Randomized design, adequate blinding and allocation concealment, clear reporting of outcomes, low risk of bias for incomplete outcome data.
Yurko-Mauro K et al., 2010 [8]	Randomized, double-blind, placebo-controlled study	24 weeks	Healthy elderly with ARCD	900 mg/d DHA or placebo	CANTAB Paired Associate Learning (PAL), Verbal Recognition Memory	Improvement in memory functions with DHA; no effect on working memory or executive functions.	DHA supplementation improves learning and memory function in ARCD.	Low risk: Well-conducted randomization and blinding, low risk of bias in reporting and data completion.
Phillips MA et al., 2015 [9]	Randomized, double-blind, placebo-controlled study	4 months	CIND or AD	Omega-3 PUFAs (600 mg EPA and 625 mg DHA per day) or placebo (olive oil)	Cognition and mood	No beneficial effects on cognition or mood; increased plasma levels of DHA and EPA.	Negligible benefit of omega-3 PUFA supplementation for cognitive impairment and dementia.	Low risk: Randomized design, proper blinding, minimal attrition, no selective reporting.
Chhetri JK et al., 2018 [10]	Randomized, controlled trial	3 years	Older subjects with increased CAIDE scores	Multi-domain intervention with or without n-3 PUFA	Cognitive tests (FCSRT, MMSE)	Improvements in memory and orientation with multi-domain intervention combined with n-3 PUFA.	Multi-domain strategies, especially with n-3 PUFA, benefit high-risk dementia subjects.	Moderate risk: While randomized, the multi-domain nature may introduce performance bias. Some concerns about incomplete reporting of certain outcomes.
Arellanes IC et al., 2020 [11]	Randomized, placebo-controlled clinical trial	6 months	Cognitive impairment	Vitamin B complex and DHA 2,152 mg/d or placebo	CSF DHA levels, MRI hippocampal volume, cognitive measures	Significant increases in CSF DHA and EPA levels; no significant changes in brain volumes or cognition.	Higher doses of DHA may be necessary for brain bioavailability, particularly in APOE4 carriers.	Low risk: Strong randomization, blinding, and allocation concealment. Comprehensive reporting, minimal risk of bias in outcome data.
Lee LK et al., 2013 [12]	Randomized, double-blind, placebo-controlled trial	12 months	Elderly with MCI	Concentrated DHA fish oil or placebo	Memory, psychomotor speed, executive function, attention, visual-constructive skills	Significant improvements in memory functions with fish oil.	Fish oil may improve memory function in MCI subjects; further research recommended.	Low risk: Properly conducted randomization and blinding, clear and complete outcome reporting.
Zhang YP et al., 2018 [13]	Randomized, double-blind, placebo-controlled trial	24 months	MCI	DHA 2 g/day or placebo	Cognitive function and Aβ-mediated autophagy	Improvements in cognitive function and reduced Aβ levels; increased levels of autophagy markers.	DHA supplementation improves cognitive function and autophagy in MCI.	Low risk: Adequate randomization, proper blinding, low attrition rates, comprehensive reporting.
Eriksdotter M et al., 2015 [14]	Randomized, placebo-controlled trial	6 months	Mild to moderate AD	2.3 g ω3 FA or placebo	Plasma FA profiles, cognition, ADAS-cog, MMSE	Preservation of cognitive functioning associated with higher ω3 FA plasma levels.	Plasma levels of ω3 FA correlate with cognitive preservation; body weight-adjusted doses recommended.	Low risk: Well-designed trial with proper blinding and randomization, low risk of bias from incomplete data or selective reporting.
Chiu CC et al., 2008 [15]	Randomized, double-blind placebo-controlled study	24 weeks	Alzheimer's disease and MCI	Omega-3 PUFAs 1.8 g/day or placebo	CIBIC-plus, ADAS-cog	Improvement in CIBIC-plus for the treatment group; significant improvement in ADAS-cog for participants with MCI.	Omega-3 PUFAs may improve clinical conditions; further studies with larger samples recommended.	Moderate risk: Potential for performance bias due to short duration and small sample size.
Zhang YP et al., 2017 [16]	Randomized, double-blind, placebo-controlled trial	12 months	MCI	DHA 2 g/day or placebo	Cognitive function, hippocampal volume	Significant improvements in cognitive function and hippocampal volumes in the DHA group.	DHA supplementation can improve cognitive function and reduce hippocampal atrophy in MCI.	Low risk: Strong methodology, low attrition, properly randomized and blinded, no selective reporting detected.

Discussion

The systematic review synthesizes findings from a series of studies examining the impact of omega-3 fatty acids, specifically DHA, on cognitive functions and brain volume in populations diagnosed with MCI or AD. A critical analysis of randomized, double-blind, placebo-controlled trials, such as those conducted by Quinn JF et al. [7] and Yurko-Mauro K et al. [8], reveals mixed results concerning the efficacy of DHA supplementation. While some studies, like that by Yurko-Mauro K et al. [8], reported improvements in memory functions in elderly individuals with age-related cognitive decline (ARCD), others, such as both trials by Quinn JF et al. [7], found no significant effect of DHA on cognitive or functional decline in Alzheimer's patients. This variability underscores the complexity of neurodegenerative conditions and the potential differential impact of DHA based on the stage of cognitive impairment and specific cognitive domains affected [17].

Further exploration within this review highlights studies, such as those by Zhang YP et al. [13] and Lee LK et al. [12], which demonstrated notable improvements in cognitive functions and reductions in biomarkers associated with neural degradation in MCI patients following DHA supplementation. These findings are complemented by Chhetri JK et al. [10], who noted cognitive enhancements from a multi-domain approach including n-3 PUFA supplementation in elderly subjects with elevated dementia risk scores. In contrast, studies like Phillips MA et al. [9] observed negligible benefits, suggesting that the role of omega-3 fatty acids may be more nuanced, possibly influenced by factors such as baseline nutrient status, genetic predispositions (e.g., APOE4 carriers, as noted by Arellanes IC et al. [11], and the specific formulations and dosages of the supplements used. This review emphasizes the need for further targeted research to optimize intervention strategies, potentially exploring personalized medicine approaches in the management of cognitive decline.

The findings of our systematic review align with and diverge from existing literature in several notable ways, reflecting the ongoing debate regarding the efficacy of omega-3 fatty acid supplementation in the management of cognitive decline. Studies like those conducted by Yurko-Mauro K et al. [8] and Lee LK et al. [12] support earlier research indicating that DHA can enhance memory functions in older adults with cognitive impairment, similar to conclusions drawn in earlier studies such as those highlighted by Freund-Levi et al. [18]. These positive outcomes echo the neuroprotective theory postulated in earlier epidemiological studies suggesting that higher dietary intake of omega-3 fatty acids correlates with reduced rates of cognitive decline.

Conversely, studies by Quinn JF et al. [7] and Phillips MA et al. [9] reported no significant benefits of DHA supplementation, a finding consistent with the MIDAS (Memory Improvement with Docosahexaenoic Acid Study) trial, which also observed limited cognitive improvement from DHA in populations with mild cognitive impairments [4]. Quinn et al. conducted a long-term, 18-month trial with a high DHA dosage (2 g/day) in participants with mild to moderate Alzheimer's disease (AD). Despite the extended duration and sufficient dose, no significant effects on cognitive decline, functional outcomes, or brain atrophy were observed. This may suggest that DHA supplementation may be less effective in later stages of AD, where neuronal damage is more advanced and synaptic loss may be too severe to reverse with DHA alone. The advanced neurodegenerative state of AD might require earlier intervention to harness DHA's neuroprotective potential.

Phillips et al. [9] conducted a shorter, 4-month study with a lower DHA dosage (625 mg/day) combined with EPA, targeting participants with Cognitive Impairment No Dementia (CIND) or AD. Although the supplementation raised plasma levels of DHA and EPA, it did not translate into cognitive improvements, possibly due to the short intervention period and lower dosage. The inclusion of both CIND and AD populations further complicates comparisons, as DHA may be more beneficial in earlier stages of cognitive decline, such as MCI. These findings underscore the need for longer interventions, higher dosages, and earlier stages of cognitive impairment for DHA supplementation to show potential benefits.

Additionally, variability in study design and intervention specifics such as the form and dosage of omega-3 fatty acids used, duration of the supplementation, and adherence levels across study participants can significantly affect outcomes [19,20]. For instance, higher doses over longer periods might be necessary to see discernible benefits, as hinted by the improved outcomes in studies administering higher doses of DHA [21]. Genetic factors, particularly the presence of APOE ε4 allele, might also modulate the response to omega-3 fatty acids, with some research suggesting that APOE ε4 carriers metabolize these fatty acids differently, potentially diminishing the neuroprotective effects observed in non-carriers [22].

This systematic review boasts several strengths, including a rigorous methodology aligned with PRISMA guidelines, ensuring a structured and reproducible approach to data collection and analysis. The comprehensive literature search spanned multiple databases, capturing a broad spectrum of studies to support an extensive synthesis of the current evidence regarding omega-3 fatty acid supplementation in cognitive decline. Additionally, the inclusion criteria were carefully designed to consider only high-quality studies, such as randomized controlled trials, which lend a high level of evidence to the review's conclusions. However, the review is not without limitations. Notable among these is the heterogeneity in study designs, populations, interventions, and outcomes, which complicates the aggregation of data and may mask the subtle effects of DHA supplementation on specific cognitive outcomes. Some studies included had relatively small sample sizes and short follow-up periods, which can limit the ability to detect long-term effects and may contribute to variability in outcomes. Furthermore, not all studies maintained blinding procedures, introducing potential bias into the results. The absence of certain types of studies, such as those exploring different stages of cognitive impairment or varying doses of supplementation, also restricts the conclusions that can be drawn from the review.

The findings of this systematic review have significant implications for clinical practice, particularly in the management and treatment of cognitive decline associated with MCI and AD. The varying results underscore the potential of omega-3 fatty acids, specifically DHA, in cognitive health, suggesting that early intervention could be beneficial for at-risk populations [23]. Clinicians should consider the stage of cognitive impairment when recommending DHA supplementation, as the evidence suggests more pronounced benefits could be realized in the earlier stages of cognitive decline [24]. Furthermore, given the safety profile of omega-3 fatty acids and the minimal risk associated with their supplementation, they could be recommended as a proactive therapeutic option to enhance cognitive resilience, particularly among those with a known risk of developing MCI or AD [25].

Building on these findings, healthcare providers might also consider integrating regular cognitive and nutritional assessments into the routine care of elderly patients. Such assessments could help identify individuals who might benefit most from targeted nutritional interventions, including omega-3 fatty acid supplementation. For instance, introducing DHA supplements could be particularly advised for individuals showing early signs of cognitive decline or those who have dietary deficiencies in omega-3 fatty acids [26]. Additionally, the review highlights the need for further research into personalized dosages and formulations of DHA to maximize therapeutic outcomes, suggesting that future clinical protocols could include genetically tailored treatments based on individual metabolic profiles and risk factors, such as APOE ε4 carrier status. These considerations could pave the way for more nuanced and effective management strategies in the ongoing fight against cognitive decline, potentially slowing the progression of conditions like MCI and AD [27].

The systematic review highlights several areas requiring further research to clarify the impact of omega-3 fatty acids, particularly DHA, on cognitive decline. A significant gap identified is the optimal timing and dosage of DHA supplementation, which varies widely across the studies reviewed. Future research should focus on determining the most effective timing and therapeutic dose of DHA for different stages of cognitive decline, considering factors such as age, baseline cognitive status, and genetic risk factors like APOE ε4 allele presence [28]. Additionally, longitudinal studies that track cognitive changes over extended periods could provide valuable insights into the long-term effects of omega-3 supplementation and its potential to alter the trajectory of cognitive decline or delay the onset of Alzheimer's disease [25].

Moreover, there is a need for larger-scale, multicenter randomized controlled trials to enhance the generalizability of the findings. Such studies should incorporate standardized measures of cognitive function and brain imaging techniques to objectively assess changes in brain structure and function over time. The inclusion of diverse populations in these studies is also crucial to understanding the differential impacts of omega-3 supplementation across various ethnic and genetic backgrounds. Meta-analyses combining data from these new, robust studies with existing research could help resolve current inconsistencies in the literature and provide more definitive guidance for clinical practice. By addressing these specific gaps and recommendations, future research could significantly advance our understanding of the potential role of omega-3 fatty acids in managing and preventing cognitive decline.

## Conclusions

This systematic review offers valuable insights into the intricate relationship between omega-3 fatty acid supplementation, particularly DHA, and cognitive decline in individuals with MCI and Alzheimer's disease. While the findings indicate potential cognitive benefits, especially in the early stages of impairment, the overall results are mixed due to variations in study designs, dosages, and participant populations. Nevertheless, the review underscores the importance of early DHA intervention as a promising approach to enhancing cognitive function, particularly in at-risk groups. It adds to the growing body of research by emphasizing the need for more personalized, targeted studies and identifying gaps in current methodologies. This review not only advances our understanding of the potential role of omega-3 fatty acids in cognitive health but also lays a solid foundation for future research, with the ultimate goal of optimizing prevention and treatment strategies for cognitive decline.

## References

[1] Chitre NM, Moniri NH, Murnane KS (2019). Omega-3 fatty acids as druggable therapeutics for neurodegenerative disorders. CNS Neurol Disord Drug Targets.

[2] Calon F, Cole G (2007). Neuroprotective action of omega-3 polyunsaturated fatty acids against neurodegenerative diseases: evidence from animal studies. Prostaglandins Leukot Essent Fatty Acids.

[3] Thomas J, Thomas CJ, Radcliffe J, Itsiopoulos C (2015). Omega-3 fatty acids in early prevention of inflammatory neurodegenerative disease: a focus on Alzheimer's disease. Biomed Res Int.

[4] Weiser MJ, Butt CM, Mohajeri MH (2016). Docosahexaenoic acid and cognition throughout the lifespan. Nutrients.

[5] Gómez-Pinilla F (2008). Brain foods: the effects of nutrients on brain function. Nat Rev Neurosci.

[6] Page MJ, McKenzie JE, Bossuyt PM (2021). The PRISMA 2020 statement: an updated guideline for reporting systematic reviews. BMJ.

[7] Quinn JF, Raman R, Thomas RG (2010). Docosahexaenoic acid supplementation and cognitive decline in Alzheimer disease: a randomized trial. JAMA.

[8] Yurko-Mauro K, McCarthy D, Rom D (2010). Beneficial effects of docosahexaenoic acid on cognition in age-related cognitive decline. Alzheimers Dement.

[9] Phillips MA, Childs CE, Calder PC, Rogers PJ (2015). No effect of omega-3 fatty acid supplementation on cognition and mood in individuals with cognitive impairment and probable Alzheimer's disease: a randomised controlled trial. Int J Mol Sci.

[10] Chhetri JK, de Souto Barreto P, Cantet C (2018). Effects of a 3-year multi-domain intervention with or without omega-3 supplementation on cognitive functions in older subjects with increased CAIDE dementia scores. J Alzheimers Dis.

[11] Arellanes IC, Choe N, Solomon V (2020). Brain delivery of supplemental docosahexaenoic acid (DHA): A randomized placebo-controlled clinical trial. EBioMedicine.

[12] Lee LK, Shahar S, Chin AV, Yusoff NA (2013). Docosahexaenoic acid-concentrated fish oil supplementation in subjects with mild cognitive impairment (MCI): a 12-month randomised, double-blind, placebo-controlled trial. Psychopharmacology (Berl).

[13] Zhang YP, Lou Y, Hu J, Miao R, Ma F (2018). DHA supplementation improves cognitive function via enhancing Aβ-mediated autophagy in Chinese elderly with mild cognitive impairment: a randomised placebo-controlled trial. J Neurol Neurosurg Psychiatry.

[14] Eriksdotter M, Vedin I, Falahati F (2015). Plasma fatty acid profiles in relation to cognition and gender in Alzheimer’s disease patients during oral omega-3 fatty acid supplementation: the OmegAD study. J Alzheimers Dis.

[15] Chiu CC, Su KP, Cheng TC (2008). The effects of omega-3 fatty acids monotherapy in Alzheimer's disease and mild cognitive impairment: a preliminary randomized double-blind placebo-controlled study. Prog Neuropsychopharmacol Biol Psychiatry.

[16] Zhang YP, Miao R, Li Q, Wu T, Ma F (2017). Effects of DHA supplementation on hippocampal volume and cognitive function in older adults with mild cognitive impairment: a 12-month randomized, double-blind, placebo-controlled trial. J Alzheimers Dis.

[17] Wen J, Satyanarayanan SK, Li A (2024). Unraveling the impact of Omega-3 polyunsaturated fatty acids on blood-brain barrier (BBB) integrity and glymphatic function. Brain Behav Immun.

[18] Freund-Levi Y, Basun H, Cederholm T (2008). Omega-3 supplementation in mild to moderate Alzheimer's disease: effects on neuropsychiatric symptoms. Int J Geriatr Psychiatry.

[19] Hong YJ, Lee JH, Choi EJ (2020). Efficacies of cognitive interventions in the elderly with subjective cognitive decline: a prospective, three-arm, controlled trial. J Clin Neurol.

[20] Michaeloudes C, Christodoulides S, Christodoulou P, Kyriakou TC, Patrikios I, Stephanou A (2023). Variability in the clinical effects of the omega-3 polyunsaturated fatty acids DHA and EPA in cardiovascular disease—possible causes and future considerations. Nutrients.

[21] Gould JF, Roberts RM, Makrides M (2021). The influence of omega-3 long-chain polyunsaturated fatty acid, docosahexaenoic acid, on child behavioral functioning: a review of randomized controlled trials of DHA supplementation in pregnancy, the neonatal period and infancy. Nutrients.

[22] Grimm MO, Michaelson DM, Hartmann T (2017). Omega-3 fatty acids, lipids, and apoE lipidation in Alzheimer's disease: a rationale for multi-nutrient dementia prevention. J Lipid Res.

[23] Castellanos-Perilla N, Borda MG, Aarsland D, Barreto GE (2024). An analysis of omega-3 clinical trials and a call for personalized supplementation for dementia prevention. Expert Rev Neurother.

[24] Zhang X, Yuan T, Chen X, Liu X, Hu J, Liu Z (2024). Effects of DHA on cognitive dysfunction in aging and Alzheimer's disease: The mediating roles of ApoE. Prog Lipid Res.

[25] Wei BZ, Li L, Dong CW, Tan CC, Xu W (2023). The relationship of omega-3 fatty acids with dementia and cognitive decline: evidence from prospective cohort studies of supplementation, dietary intake, and blood markers. Am J Clin Nutr.

[26] Lindner-Rabl S, Wagner V, Matijevic A, Herzog C, Lampl C, Traub J, Roller-Wirnsberger R (2022). Clinical interventions to improve nutritional care in older adults and patients in primary healthcare - a scoping review of current practices of health care practitioners. Clin Interv Aging.

[27] Yassine HN, Braskie MN, Mack WJ (2017). Association of docosahexaenoic acid supplementation with Alzheimer disease stage in apolipoprotein E ε4 carriers: a review. JAMA Neurol.

[28] Derbyshire E (2018). Brain health across the lifespan: a systematic review on the role of omega-3 fatty acid supplements. Nutrients.

